# A more natural follicle culture system: Detailed steps of In Vitro 3D follicle culture with alginate gel

**DOI:** 10.1016/j.mex.2024.102756

**Published:** 2024-05-24

**Authors:** Yang Zhang, Zhe Zhang, Xiaoqiang Sheng

**Affiliations:** a Center for Reproductive Medicine and Obstetrics and Gynecology, Nanjing Drum Tower Hospital, Affiliated Hospital of Medical School, Nanjing University, Nanjing, China; b Center for Molecular Reproductive Medicine, Nanjing University, Nanjing 210008, PR China; c Center for Reproductive Medicine, The Second Affiliated Hospital and Yuying Children's Hospital of Wenzhou Medical University, Wenzhou, Zhejiang, China

**Keywords:** *In-vitro* 3D follicle culture, Follicle development, Alginate gel, 3D culture, Microenvironment

## Abstract

Follicle culture is a process of dividing follicle unit structures from ovaries for continued culture *in vitro* in an incubator, which simulates the *in vivo* environment. Alginate gel is the most stable and most convenient 3D material currently used in follicle culture. We performed *in vitro* follicle culture following the standard operating procedure recommended by the Follicle Handbook and we have summarized our experience and skills in details. Through several experiments, we found only follicles tightly surrounded by theca cells can grow healthily until the preovulatory stage. In addition, the hardness of alginate gel is crucial for constructing the 3D culture system, and selecting appropriate tools can reduce damage to the alginate gel and shorten the time follicles are exposed to room temperature. Our detailed operation improves bioavailability and provides a more natural environment for the entire process of follicular growth.

• Alginate gel is still the most suitable 3D material used for *in vitro* follicle culture.
• Follicle integrity and the hardness of alginate gel are the keys for *in vitro* culture.
• Detailed operation steps better protect the follicular microenvironment and improve bioavailability.

## Graphical abstract

**Figure:**
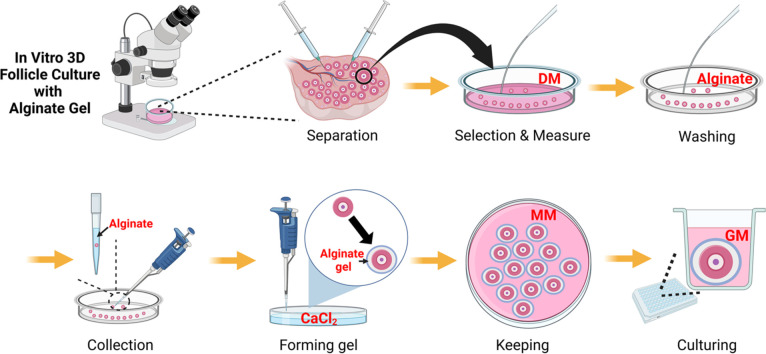

Specifications Table

**Table:** 

Subject area:	Medicine and Dentistry
More specific subject area:	Follicle culture
Name of your method:	*In-vitro* 3D follicle culture
Name and reference of original method:	Woodruff and Shea Labs. (2014). Follicle Handbook. Reproduction 145(1):19–32
Resource availability:	Alginate gel, Pasteur pipettes, 96-well culture plate

## Method details

### Background

*In vitro* follicle culture involves mechanically dissecting ovaries into follicles of different sizes, selecting the early secondary stage follicles and adding small molecules to encourage them to grow *in vitro* until the preovulatory stage [1], [2], [3], which can simulate the entire process of follicle development and maturation *in vivo* [4,5]. Since the end of the last century, the emergence of 2D culture systems has provided great assistance in studying follicular growth and development, but they limit the growth of individual follicular structures and shorten the follicular lifespan [6]. With the ongoing development of culture systems, it has been found that 3D culture systems can better simulate the ovarian environment *in vivo*, and water-soluble alginate gel is undoubtedly a preferred biomaterial [3,7]. Alginate gel can undergo ion exchange in calcium solution for thermally irreversible gel formation [8]. Compared with other biomaterials, the elasticity and strength of alginate gel better simulate the mechanical conditions for follicular growth in the ovarian medulla and cortex *in vivo* [7,[9], [10], [11]. Alginate gel makes *in vitro* follicle 3D culture an advantageous method for analyzing follicle microenvironments [12].

A successful 3D culture system involves several important factors, including selecting follicles of an appropriate size, preserving the integrity of the follicular structure, ensuring stable culture conditions and producing gels with appropriate hardness. These steps have been described in operation manuals and the literature, and skillful operation is crucial. In the past, we used to increase the number of isolated follicles to compensate for experimental losses caused by mechanical damage, temperature changes, gel dissolution and gel hardness, which also increased the loss of biological materials, prolonged the operation time and greatly reduced bioavailability [13].

Based on the guidelines provided in the Follicle Handbook [11,12], we described and standardized each step in the operation process in as much detail as possible, which significantly improved the follicular survival rate in the culturing process [14]. Early secondary follicles with a diameter of at least 110 µm can grow steadily *in vitro* for 10–12 days until they reach a diameter of over 400 µm and form follicular cavities. The survival rate of follicles isolated *in vitro* can reach over 90 %, and follicles cultured *in vitro* can also be fertilized and form blastocysts, although the blastocyst formation rate is still low compared with that of fertilization *in vivo.* However, standardizing the operation does provide significant benefits.

## Method details

### Materials

**Fig. 1:**
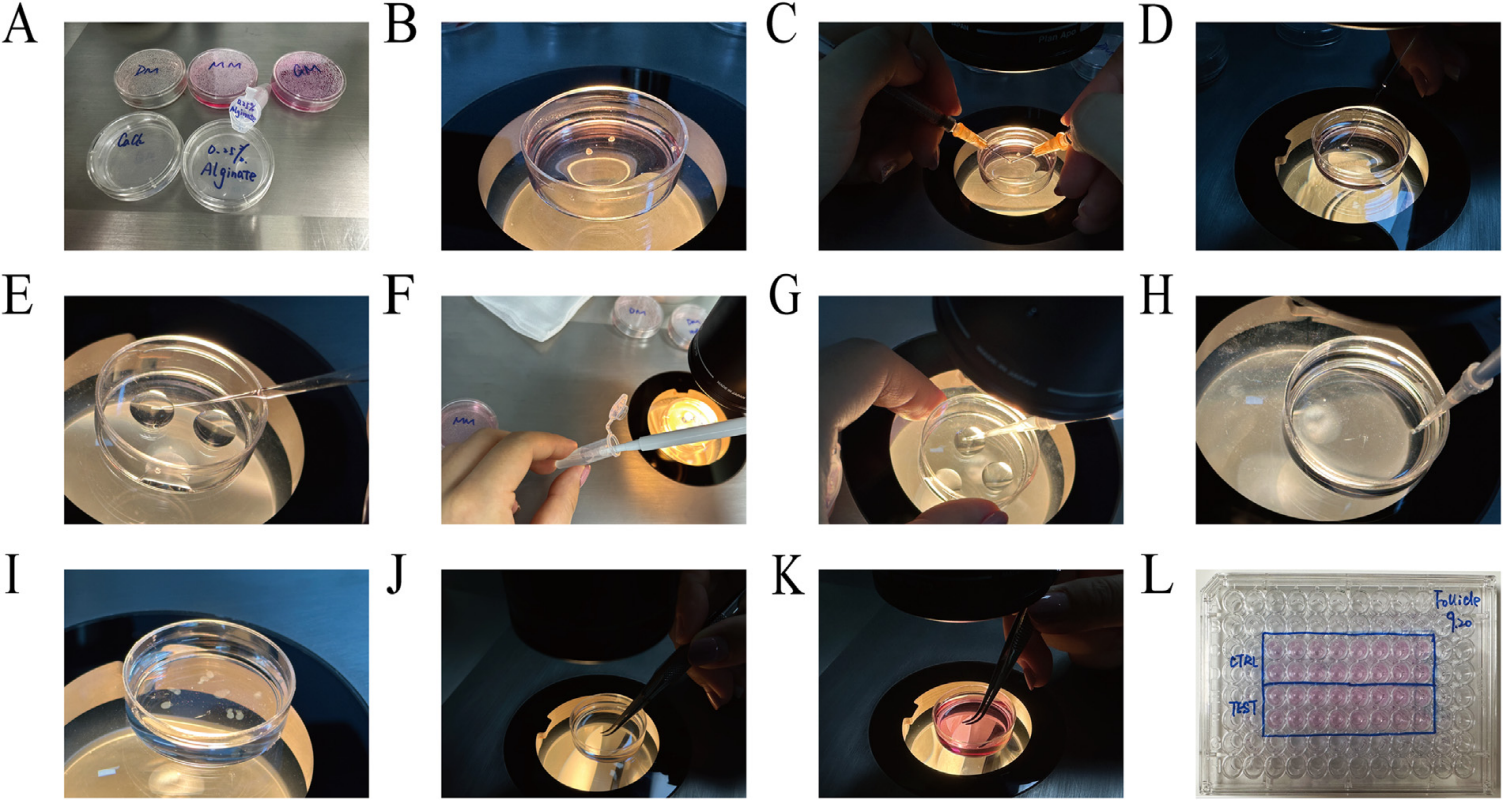
Method details of *in vitro* 3D follicle culture. (A) Reagent preparation for *in vitro* follicle culture. (B) Collect ovaries and place them in the DM dish. (C) Use 1 mL syringes or insulin injection needles to separate follicles. (D) Select early secondary follicles with the appropriate diameter and place them in a new DM dish. (E) Successively transfer follicles into an alginate washing dish and an alginate gel dish. (F) Place a small amount of 0.25 % alginate solution into a 1.5 mL eppendorf tube, and aspirate 5 µL liquid. (G) Aspirate follicles into the pipette tip. (H) Submerge the pipette tip into CaCl_2_ solution and quickly eject the liquid with the follicle. (I) A light milky gel will form in the CaCl_2_ solution. (J) Transfer alginate gel from the CaCl_2_ dish to the MM dish by using nontoothed microforceps. (K) Transfer follicles into a 96-well plate with toothed tissue forceps. (L) Place one mass of gel in one well and mark with culture information on the 96-well plate.

The equipment and reagents needed for *in vitro* 3D follicle culture are as follows (all reagents are listed in the Follicle Handbook [10,11], and can be stored at 4 °C for only 2 weeks). The instruments include tissue scissors, dentate tissue forceps, 35 mm dishes, 96-well cell plates, 1 mL syringes, stereoscopes, eyepieces with scales, straight microforceps, nontoothed microforceps, Pasteur pipettes, and transfusion guns. The reagents include M2 medium, dissection medium (DM), maintenance medium (MM), growth medium (GM), CaCl_2_ solution, and 0.25 % alginate solution (Fig. 1A). To ensure aseptic operation during follicle culture, all instruments must be sterilized, and the reagents should be filtered in advance.

### Animals

All animal experiments were conducted under the guidance of the Laboratory Animal Management Committee (Jiangsu Province, China) and approved by the Institutional Animal Care and Use Committee of Nanjing Drum Tower Hospital (SYXK 2021–0509). Mice should be 13∼15 days old mice with healthy hair and weight, as differences in breastfeeding in one litter decrease the primordial follicle reserve [15,16]. The follicles in the early secondary stage are the most abundant of 13–15-day-old mice, so more follicles (target diameter 110–130 µm) can be isolated for culture *in vitro* [17]. The follicles in younger mice are mostly primary follicles, which are not difficult to separate, but they are difficult to grow *in vitro*. Due to differences in growth and development rates, follicles in older mice are not uniform, and there is more fibrotic tissue in the ovaries, which makes the operation too difficult.

### Follicle separation

1. Preheat the DM dishes on the 37 °C hot stage, collect the ovaries and place them in the DM liquid (Fig. 1B).
  Tip 1: It is recommended to collect ovaries when follicle isolation is needed. Do not place ovaries in the DM dishes for too long, because long-term immersion will increase viscosity during separation.
2. Use 1 mL syringes or insulin injection needles to separate follicles (Fig. 1C). We recommend flipping the ovary through the ovary “door” from the medulla side to the cortical side using the needle. After dividing the ovarian tissue into small blocks, use one needle to gently fix the tissue blocks, and use the other needle to prod the stroma around the follicles to move or pull, ultimately separating an intact follicle. We advised keeping the follicular structure as intact as possible. This means that the follicle needs to retain one circle of theca cells and even a few attached stromal components, which can guarantee that follicles grow stably on the second day (Fig. 2A). Follicles with damaged or missing theca cells will die on the second day (Fig. 2B), so it is advisable to use the back side of the needle during separation. Make sure the needle bevel is perpendicular to the bottom of the dish when pulling or cutting the stroma to prevent plastic filaments from adhering to the follicles.

**Fig. 2:**
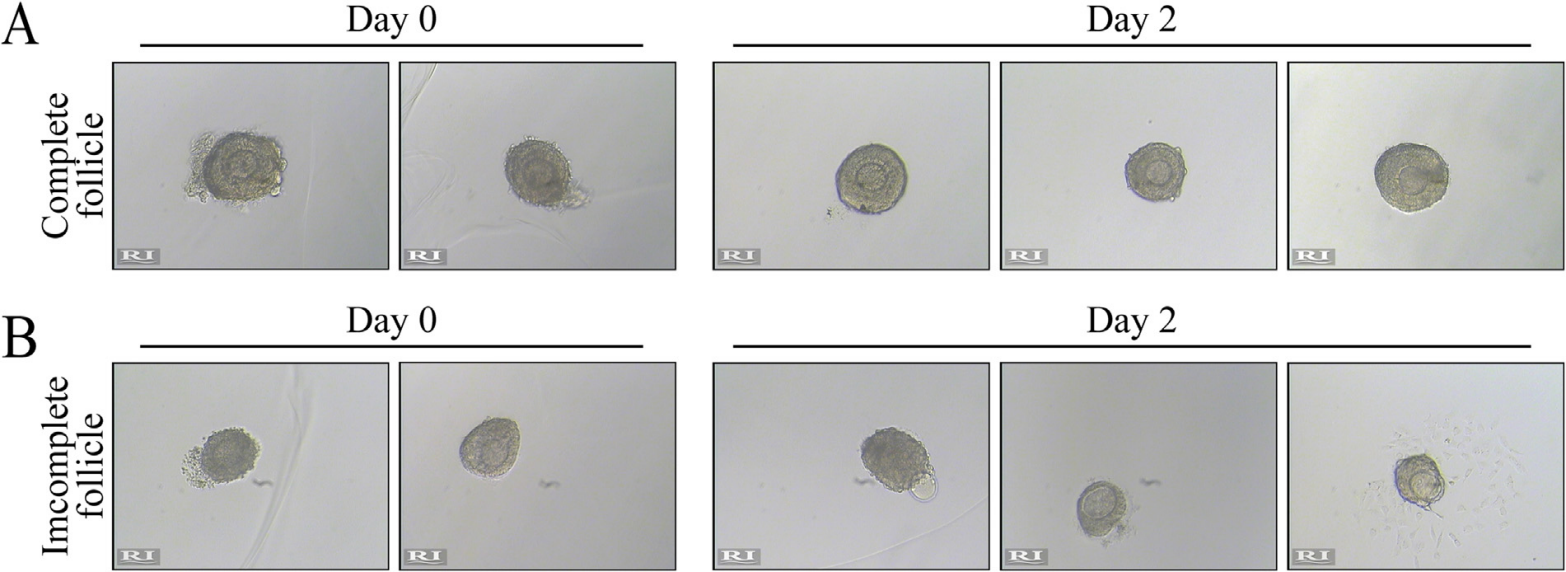
Only complete follicles with theca cells and theca components can develop normally. (A) A follicle retaining one circle of theca cells, possibly with a few stromal components attached, can grow stably on the second day. (B) Follicles with damaged or missing theca cells will die on the second day.
  Tip 2: According to the Follicle Handbook [10], it is recommended to use collagenase and DNase to digest ovaries; however, digestion with enzymes will increase the viscosity of stromal components, making them adhere easily to the needle or Pasteur pipette, which will cause the loss of follicles. We still recommend separating follicles through mechanical cutting.
  Tip 3: The ovarian cortex is more compact than the medulla and requires more cutting force to separate follicles, which may damage the theca cells around follicles. Therefore, we recommend flipping ovaries from the medulla side to the cortical side and starting to cut from the medulla side.
3. After cutting, select early secondary follicles with appropriate diameters and keep them in a new DM dish (Fig. 1D). In the first operation, we recommend selecting follicles of different sizes and measuring their diameters in the RI software, then remeasuring them under the stereoscope and estimating the scale of each follicle through the eyepiece. In later experiments, use the eyepiece with scale to directly select follicles to save time. For example, in our experiments, 130–150 µm of follicles is 8–9 scale marks of the eyepiece.
  Tip 4: Pre-wet the Pasteur pipette with DM liquid in advance and retain some fluid before transferring follicles because the residual stroma around follicles easily adheres to the dry inner wall of the Pasteur pipette.
  Tip 5: The growth and development rates of follicles with different initial diameters are not completely uniform, and the larger the initial diameter is, the faster the growth rate. To ensure the authenticity and repeatability of the experiment, it is necessary to minimize the range of initial follicle diameters as much as possible.
  Tip 6: The eyepiece with scale can only be used as a tool for roughly estimating the diameter to save operation time. After placing in a 96-well plate, the diameter needs to be remeasured for recording by the RI software.
4. Prepare an alginate washing dish and an alginate gel dish (the alginate solution is balanced at room temperature in advance), make several 0.25 % alginate droplets at the bottom of the dish, transfer the follicles into the washing droplets with a Pasteur pipette, and then transfer them into the alginate gel droplets (Fig. 1E) after washing 2–3 times.
  Tip 7: Alginate washing dish and alginate gel dish can be placed on the hot plate.
  Tip 8: Pre-wet the Pasteur pipette with 0.25 % alginate solution or prepare the new Pasteur pipette before transferring follicles to prevent the follicles from adhering to the inner wall of the pipette.
  Tip 9: The Follicle Handbook [10] recommends using 0.5 %−1 % alginate solution to make the gel, and we tried making the gel using 0.25 %, 0.35 %, 0.5 % and 1 % alginate solution; however, our results showed that the gel made with 0.25 % alginate solution could appropriately prolong the gel formation time and improve the efficiency of the operation process. Moreover, 0.25 % alginate gel does not display yellowing or hardening due to oxidation after 10–12 days of *in vitro* culturing.
5. Prepare a CaCl_2_ solution dish (the CaCl_2_ solution is balanced at room temperature in advance) with as much volume as possible to ensure that the gel can float completely in the liquid. Place a small amount of 0.25 % alginate solution into a 1.5 mL Eppendorf tube, aspirate 5 µL of liquid (Fig. 1F), put the pipette tip close to the follicles, gently eject a small amount of liquid into the alginate gel droplets, and then aspirate the follicles into the pipette tip (Fig. 1G). Submerge the pipette tip into the CaCl_2_ solution (the pipette tip is vertical to the bottom of dish), try not to shake the pipette tip, and quickly eject the liquid with the follicle (Fig. 1H). A light milky gel will be formed in the CaCl_2_ solution after 2–3 min (Fig. 1I). Replace the pipette tip and aspirate the next follicle.
  Tip 10: The CaCl_2_ solution should be mixed by inversion before use to prevent solid precipitation. Do not put the CaCl_2_ dish on the hot plate during the gel-making process, because it is difficult to make gel in warm CaCl_2_ solution.
  Tip 11: Here are the advantages of changing pipette tips:
  1. The pipette tip is wet with alginate solution to prevent follicles from adhering to the inner wall of the pipette tip.
  2. The residual alginate solution on the pipette tip will also form a gel after contact with CaCl_2_, and repeated use of the same pipette tip may block the pipette tip or reduce the gel volume.
  3. It avoids follicular contamination caused by repeated use of the same pipette tip.
  Tip 12: The structure of alginate gel in CaCl_2_ solution is stable, but its hardness increases with time. Keeping the gel in CaCl_2_ for 2–3 min is our empirical recommendation; however, the duration can be adjusted according to the experimental conditions. On the one hand, it can be observed that the color of the gel turns to light milky white. On the other hand, the gel can be gently pressed with microforceps to test the hardness of the gel.
6. Transfer the formed alginate gel from the CaCl_2_ dish to the MM dish (preheat MM dish to 37 °C in advance, and use enough liquid to submerge the gel) (Fig. 1J). Use edentulous micro-forceps to transfer follicles, and store them temporarily in the incubator at 37 °C.
7. After all the follicles are wrapped in alginate gel, prepare the 96-well plate required for *in vitro* culture. The GM solution should be preheated in an incubator at 37 °C in advance, 100 µL GM solution is added to each well of the 96-well plate, and the gel containing a follicle is transferred by dentate tissue forceps (Fig. 1K). To measure the diameter of each follicle separately for recording, we suggest that one mass of gel should be placed in one well and should be marked with the culture information on the 96-well plate (Fig. 1L).
  Tip 13: The hardness of the gel soaked in MM solution will decrease, so it is easier to transfer gel using dentate tissue forceps. Be careful not to break the gel during the transfer process, which may cause the follicles to fall out of the gel during the culture process.
8. On the first day, place the 96-well plate under a microscope with a heated plate to measure the diameter of the follicle. Record the longest diameter of the follicle as the initial diameter of culture on Day 0 (Fig. 3A). In the subsequent culture process, 50 µL of GM solution in every well is changed every other day, and the diameter should be measured at Day 2, Day 4, Day 6, Day 8, Day 10 and Day 12 (Fig. 3B) to draw the growth curve (Fig. 3C). Usually, some follicles can be found forming follicular cavities on Day 6 (Fig. 3D). The diameter of the follicle on Day 10 can reach 400 µm and enter the plateau stage by degrees [13].

**Fig. 3:**
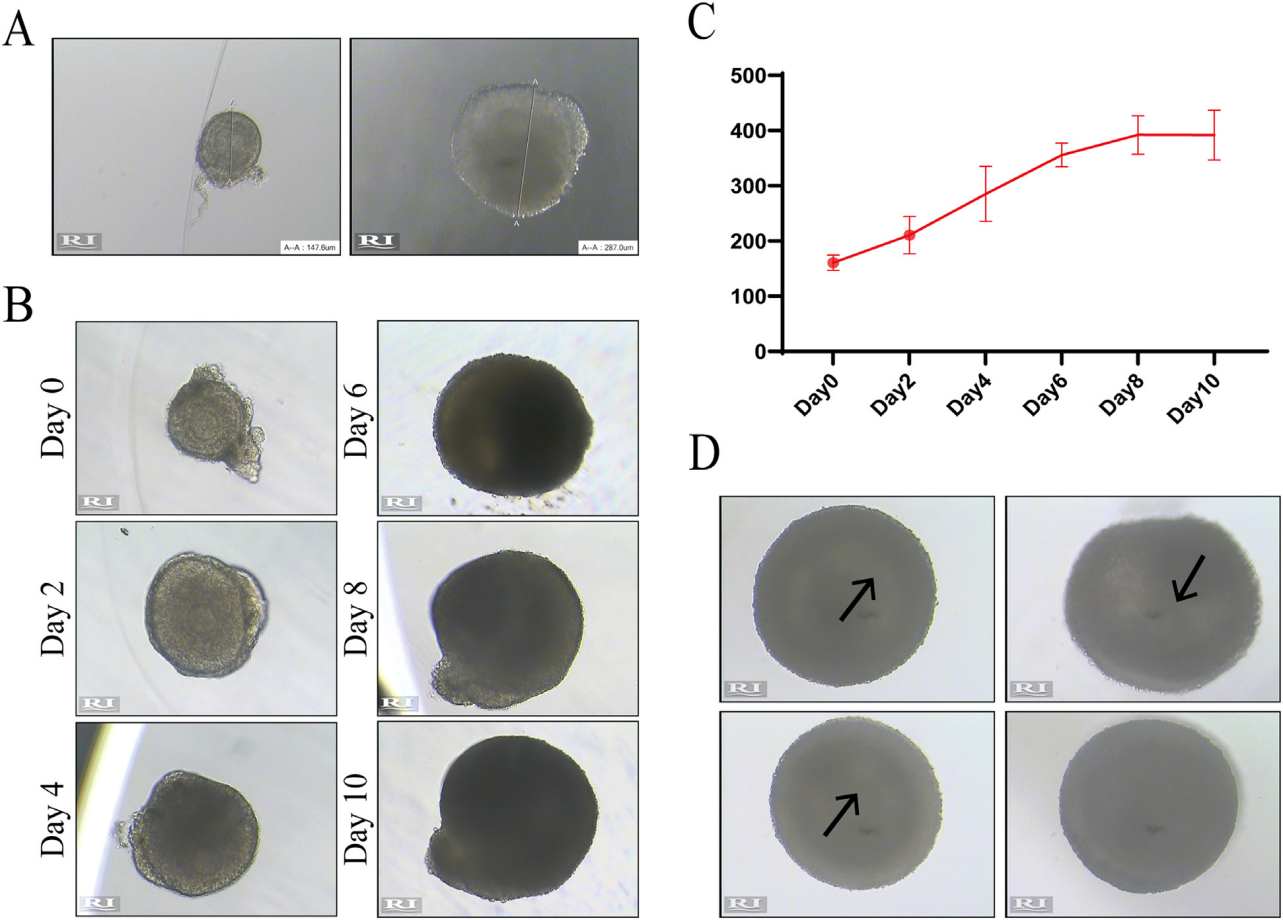
Measure the diameter with RI software to record follicle development. (A) Record the longest diameter of the follicle as the initial diameter of culturing on Day 0. (B, C) Measure follicle diameter on Day 2, Day 4, Day 6, Day 8, Day 10 and Day 12 (B) and draw the growth curve (C). (D) Follicular cavity can form on Day 6. The arrow represents the formed follicular cavity.
9. Mature preovulatory follicles can be used for *in vitro* maturation (IVM) and *in vitro* fertilization (IVF) experiments [18]. Oocytes at the GV stage can be obtained by acupuncture or incision and matured *in vitro* according to the IVM conditions provided in the Follicle Handbook [10], and IVF can also be continually carried out (Fig. 4A). However, at present, the quality of oocytes cultured *in vitro* still cannot completely replicate that obtained *in vivo.* Nearly half of the fertilized oocytes can develop into two-cell embryos (the two-cell rate is approximately 53 %), but they are essentially blocked at the four-cell embryo stage. By constantly optimizing the details of the operation, we finally increased our blastocyst rate from 0 % to 22 % (Fig. 4B), although there is still much room for further improvement.

**Fig. 4:**
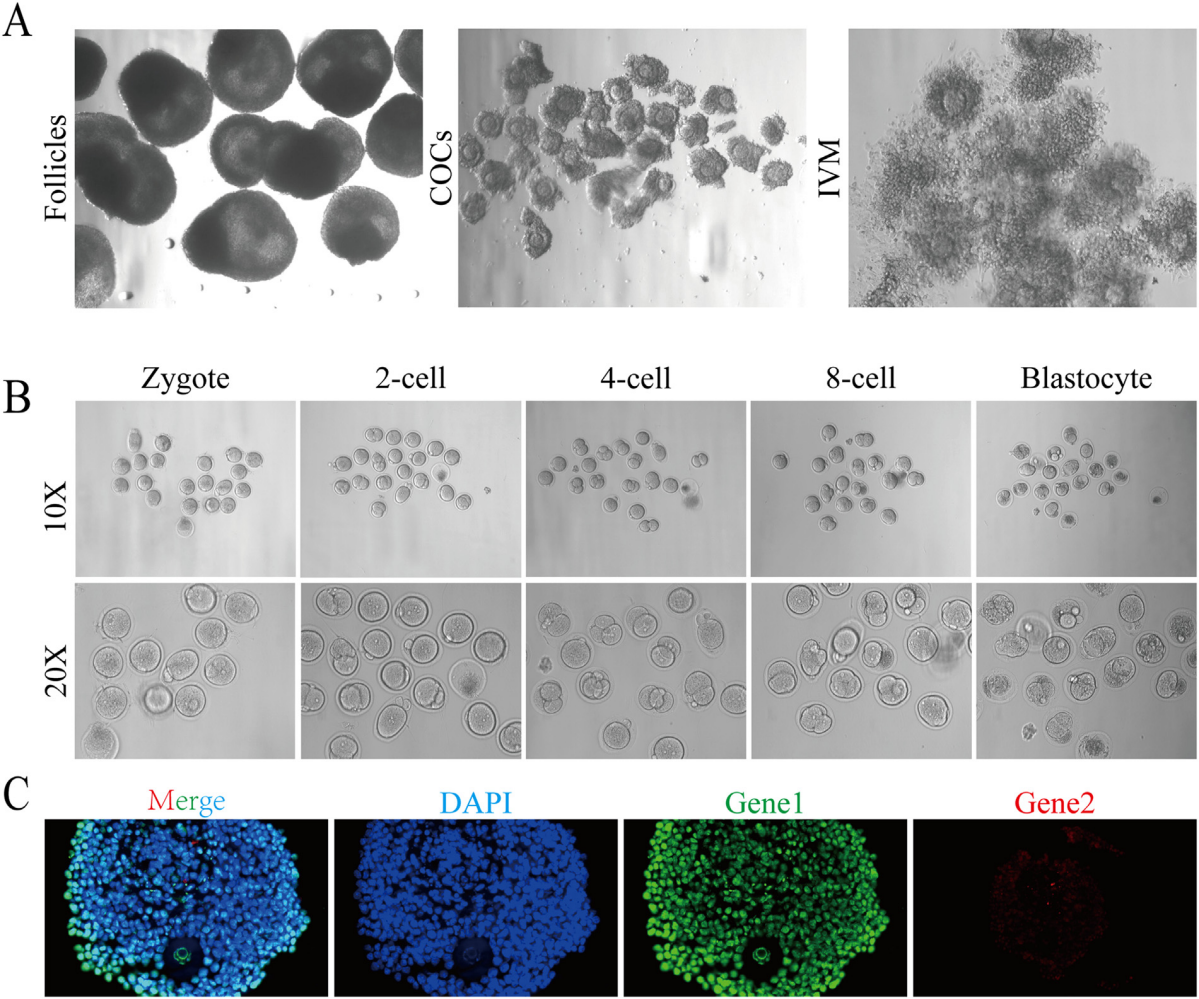
Follicles cultured by *in vitro* 3D culture system can be used in IVM, IVF and other molecular experiments. (A, B) Mature preovulatory follicles can undergo IVM (A) and IVF (B) experiments. The arrow represents the formed blastocyst. (C) Follicles cultured *in vitro* can be used in the immunofluorescence staining.
10. According to the Follicle Handbook [10], follicles cultured *in vitro* can also be used in real-time quantitative PCR (RT-qPCR), immunohistochemistry staining and immunofluorescence staining. RT-qPCR was tested using a primer pool. The volume of preovulatory follicles is still small and it is difficult to fix slices even after being wrapped with alginate gel. We also optimized the details of fixed dehydration based on the Follicle Handbook. Manual gradient dehydration was carried out in the order of 70 % ethanol–80 % ethanol–90 % ethanol–100 % ethanol-100 % ethanol-100 % ethanol: xylene = 1: 1–xylene–xylene. Keep follicles in each liquid for 2 min, and then dip the follicles in paraffin for 30 min. Finally, embed follicle in paraffin for slicing. However, it is still very difficult to obtain a complete follicular structure in the process of immunohistochemical staining and immunofluorescence staining (Fig. 4C), which is another drawback that cells for optimization in the process of *in vitro* follicle culture.

## Ethics statements

All animal experiments were approved by the Affiliated Drum Tower Hospital, Medical School of Nanjing University's Committee on the Use and Care of Animals. All operations follow appropriate animal care in the EU Directive 2010/63/EU for animal experiments.

## CRediT authorship contribution statement

**Yang Zhang:** Validation, Data curation, Writing – original draft, Writing – review & editing. **Zhe Zhang:** Validation, Data curation, Writing – original draft, Writing – review & editing. **Xiaoqiang Sheng:** Conceptualization, Methodology, Validation, Data curation, Writing – original draft, Supervision, Writing – review & editing.

## Declaration of competing interest

The authors declare that they have no known competing financial interests or personal relationships that could have appeared to influence the work reported in this paper.

## Data Availability

Data will be made available on request.
